# Researching violence against women and girls in South Sudan: ethical and safety considerations and strategies

**DOI:** 10.1186/s13031-019-0239-4

**Published:** 2019-11-21

**Authors:** Manuel Contreras-Urbina, Alexandra Blackwell, Maureen Murphy, Mary Ellsberg

**Affiliations:** 1 The Global Women’s Institute, George Washington University, 2140 G Street NW, Washington, DC, 20052 USA; 2 International Rescue Committee, 1730 M St NW #505, Washington, DC, 20036 USA

**Keywords:** Conflict, Emergencies, Ethics, Gender-based violence, Humanitarian, Research, Safety, South Sudan, Violence against women and girls (VAWG)

## Abstract

**Background:**

Globally, it is estimated that at least one out of every three women experiences violence by an intimate partner and/or non-partner throughout their lifetime. Women and girls are at even higher risk of violence in conflict and humanitarian crises. Although effort has expanded to build rigorous evidence and research on violence against women and girls (VAWG) among conflict-affected populations, methodological and ethical challenges remain. Basic ethical research practices are more challenging in conflict-affected populations and therefore require supplementary protections. While it is important to follow international ethical guidelines, in practice it is sometimes difficult depending on the setting. The aim of this paper is to present the main ethical challenges that occur when conducting research on VAWG in conflict and humanitarian settings, as well as potential strategies to address these challenges, based on a recent study carried out in South Sudan in 2016.

**Case presentation:**

This paper provides an analysis utilizing the World Health Organization guidelines on doing research on VAWG and in conflict and humanitarian settings. The paper analyses four main components: the first component is a risk-benefit assessment, which includes identifying the research gap and evaluating feasibility with local stakeholders. The second component is a methodological and conceptual approach, which involves both local stakeholders and external experts in order to develop flexible methods that can be used in a volatile conflict setting. The third component is safety considerations, which emphasizes the importance of collaborating with a partner with strong local networks. The last component is analysis and research uptake. This component describes the importance of developing different research products, and disseminating them in a way to ensure they would be relevant and minimize any risks to the participants.

**Conclusions:**

The study in South Sudan provided an excellent opportunity to put into practice the international ethical guidelines to carry out research on VAWG in conflict and humanitarian settings. The study enabled the research team to reflect on the guidelines and develop strategies to cope with new methodological and ethical challenges that arose in this complex setting, adapting the guidelines, as necessary. It demonstrated the necessity of developing a strong yet adaptable methodology with multiple alternative plans to solve any safety or ethical issues that occur throughout the entirety of the study. It also revealed the importance of collaborating with an implementing partner and gathering input from both local and international stakeholders on research design, analysis and uptake. Most importantly, the study in South Sudan emphasized that this type of complex research requires significant planning, in addition to substantial financial and human resources. Donor buy-in and flexibility is therefore essential.

## Background

### Introduction

Globally, it is estimated that at least one out of every three women experiences violence or abuse at the hands of an intimate partner or non-partner throughout their lifetime [1]. Women and girls are at even higher risk of violence in conflict and humanitarian crises due to a number of factors, including displacement, the breakdown of social structures, a lack of law enforcement, the potential further entrenchment of harmful gender norms, and the loss of livelihood opportunities for both men and women in the community, among others [2].

In recognition of the need to address this issue, the international community has expanded their efforts to build rigorous evidence and research on the different types of violence against women and girls (VAWG) among conflict-affected populations. However, these contexts present a number of methodological challenges, and much of the research is not comparable due to the varied application of methods, study outcomes and definitions. Moreover, there is a lack of consistency regarding the approaches to ensuring the confidentiality and safety of participants and researchers.

Attention to ethics is essential when implementing research with human subjects. Basic ethical principles, as set forth by the Belmont Report [3], are evoked for all research pertaining to human subjects. Given the sensitive nature of the topic of VAWG and the risks it presents for the physical and psychological safety of both participants and members of the research team, the World Health Organization (WHO) published the guidelines “Putting Women’s Safety First: Ethical and Safety Recommendations for Research on Violence against Women” (1999) [4]. The guidelines present eight key ethical and safety principles for research on VAWG that establish standards for procedures including maintaining confidentiality, minimizing underreporting, fieldworker training, referral systems and presentation of findings. The WHO guidelines are considered a gold standard for conducting research on VAWG and have been adopted by the vast majority of institutions that carry out or fund research on violence.

### Ethical framework for researching violence against women and girls in conflict and humanitarian settings

Research on sensitive topics conducted among displaced or other conflict-affected populations presents additional ethical and safety challenges, as the populations under investigation have overlapping vulnerabilities that compound the risks of conducting research with these groups.

Therefore, the WHO adapted their guidelines for emergency settings and developed “Ethical and safety recommendations for researching, documenting and monitoring sexual violence in emergencies” (2007) [5].

**Table:** 

WHO ethical and safety recommendations for researching, documenting and monitoring sexual violence in emergencies (2007)	
1. The benefits to participants or communities of documenting sexual violence must be greater than the risks to participants and communities. 2. Information gathering and documentation must be done in a manner that presents the least risk to participants, is methodologically sound, and builds on current experience and good practice. 3. Basic care and support for survivors/victims must be available locally before commencing any activity that may involve individuals disclosing information about their experiences of sexual violence. 4. The safety and security of all those involved in information gathering about sexual violence is of paramount concern and in emergency settings in particular should be continuously monitored. 5. The confidentiality of individuals who provide information about sexual violence must be protected at all times. 6. Anyone providing information about sexual violence must give informed consent before participating in the data gathering activity. 7. All members of the data collection team must be carefully selected and receive relevant and sufficient specialized training and ongoing support. 8. Additional safeguards must be put into place if children (i.e. those under 18 years) are to be the subject of information gathering.	

These recommendations provide general guidance for collecting data on sexual violence in high-risk contexts; however, they are applicable for research on other sensitive topics, including other types of VAWG that occur during emergencies. They mainly focus on the importance of protecting participants in the study. As part of research design, the WHO guidelines emphasize the need to evaluate the value of the research and to weigh potential costs and benefits. Additionally, they highlight the importance of employing a methodology that is grounded in experience and good practice and that presents the least risk to participants. They also emphasize the supplementary mechanisms that should be in place to ensure the confidentiality, privacy, voluntariness and safety of participants, including additional considerations during informed consent. The guidelines provide specific considerations around respect for participants given the potentially threatening and traumatic nature of VAWG as a subject, including referrals to quality local support for survivors and the selection and training of enumerators. While these guidelines are critical to carry out research on VAWG in conflict and humanitarian settings, the application of these universal ethical standards could present several practical difficulties when implementing this type of research. In addition, some of these considerations might not be sufficient to protect individuals due the unique challenges of a conflict environment.

### This study

This paper is a case study that aims to present reflections and analysis of the main ethical challenges that occur when conducting research on VAWG in conflict and humanitarian settings, as well as potential strategies to address these challenges, based on a recent study carried out in South Sudan in 2016 [2]. The study was part of the United Kingdom Department for International Development (DfID) global program, entitled, *What Works to Prevent Violence Against Women and Girls.* The research was carried out by The Global Women’s Institute (GWI) at the George Washington University in partnership with the International Rescue Committee (IRC), CARE International UK (CIUK), and Forcier Consulting. The research team included international and national researchers representing these organizations. The research study used both qualitative and quantitative methods to understand the prevalence, types and patterns of VAWG among populations who have been affected by current and past conflicts across five sites in South Sudan.

Using the experience of the research in South Sudan, this paper seeks to contribute to efforts to improve research on VAWG in conflict and humanitarian settings while ensuring the safety of those involved (both participants and the researchers), and to make recommendations that can inform future research in similar high-risk settings.

### The context: civil and intercommunal conflict in South Sudan

In 2011, South Sudan emerged from 25 years of civil war with the central Government of Sudan and 6 years under the 2005 Comprehensive Peace Agreement (CPA) to become the world’s newest nation state [6]. However, the undercurrent of political and ethnic conflicts remained after independence, and in December 2013 violence erupted once again, sparking the South Sudanese civil war referred to as the 2013 Crisis. In spite of the establishment of a new peace agreement in 2015, civil conflict persisted across the country, and inter-communal conflicts were a continuing facet of this period of relative peace [6]. These conflicts often centered on localized tensions such as land for cattle grazing, accumulation of wealth (by means of cattle raiding) and abduction of women or children [7]. Many of these incidents triggered revenge killings from the victimized community that caused a cycle of revenge attacks, perpetuating continued insecurity. The ethnic civil conflict then re-ignited during an outbreak of violence in Juba City in July 2016. This constant unrest has eroded the education and political systems and deteriorated the local economy, leaving little to no institutional structures to deliver services or facilitate decision-making [8]. This, combined with frequent famines and other crises, has exacerbated the situation of extreme poverty and insecurity in the country, especially for women and girls.

### Methodological and ethical considerations of the study

The study used a mixed-methods approach to fill substantial gaps in understanding on the intersections of VAWG and conflict in South Sudan. The quantitative component consisted of a population-based household survey administered to a representative sample of women aged 15–64 in three locations (Juba City, Rumbek Centre and the Juba’s Protection of Civilian - POC - camps), as well as a supplemental survey for men (aged 15–64) in two locations (Juba City and Rumbek Centre). The questionnaire was based on the *WHO Multi-country Study on Women’s Health and Domestic Violence Against Women* [9] and was adapted for the unique context of South Sudan. A multi-stage cluster sampling design was used to select individual households for inclusion in the cross-sectional survey. Interviews with men and women were conducted in different clusters to ensure the confidentiality and security of participants. A systematic sampling strategy was used for household selection. A simple randomization strategy was used to select one individual in the household without replacement to avoid bias. A total of 2244 women and 481 were interviewed. For more information on the sampling frame, view the full report [2].

Qualitative data was collected in five locations in South Sudan. The qualitative data was collected with community members, key informants (non-governmental staff, government representatives, local leaders, etc.) and survivors of VAWG. In addition to the three sites included in the household survey, in-depth interviews and focus group discussions (FGDs) were also conducted in Juba County and the POC camp in Bentiu. Semi-structured, in-depth interviews were conducted with female survivors of violence who had sought and received services from IRC. The FGDs utilized participatory tools, which have been used for research on VAWG in other settings, including free-listing and open-ended stories/Venn Diagrams.

The research protocol was approved by the Institutional Review Board (IRB) of the George Washington University as well as the Technical Advisory Group (TAG) in South Sudan, which is an independent body of experts in VAWG research and programming in South Sudan made up of local, national and international practitioners representing both NGO and government structures. Permission to conduct the research was secured with appropriate authorities at national and local levels.

The research followed the WHO’s Ethical and Safety Recommendations for Researching, Documenting and Monitoring Sexual Violence in Emergencies [5]. The fieldwork team was carefully selected and trained during an interactive three-week training session held at each site, which included instruction on managing privacy, confidentiality and participant distress, as well as questionnaire and study methodology. All fieldwork supervisors and enumerators were South Sudanese. However, despite the ethical and methodological considerations made during the design of the study, challenges to follow these guidelines emerged given the situation of South Sudan at the time of the survey, and new approaches had to be developed to overcome these challenges.

## Case presentation: ethical and methodological considerations and strategies for research in conflict settings

Utilizing the WHO framework, this paper presents an analysis of the key ethical considerations and challenges that arose during the study in South Sudan and the strategies used for overcoming those challenges. This section is organized by four main components of ethical research on VAWG in conflict and humanitarian situations that addressed the main ethical challenges and dilemmas faced when doing this research: 1) risk-benefit assessment; 2) methodological and conceptual approaches; 3) safety considerations; and 4) analysis and research uptake.

### Risk-benefit assessment

The WHO guidelines highlight the importance of assessing potential harms and benefits for studies in conflict and humanitarian settings before initiating research activities. Considering the unstable situation of the country, exploring the purpose and added value of the study in South Sudan was extremely important in order to determine if the study was feasible and benefited the participants. For that reason, the research team undertook a continuous risk-benefit assessment to determine whether the study was truly necessary and feasible, and how the evidence could be used to inform policies and programs. The assessment aimed to confirm the following:

- The research was needed in this specific context and the research questions had not already been answered in previous research.
- There were national and local stakeholders and policy-makers engaged to ensure the findings would be useful for the development of actions that could potentially improve the lives of women and girls.
- The research could be conducted with minimum risks to participants, researchers and fieldworkers.
- There were referral services in place to provide essential support to participants.
- There were the means to protect the confidentiality and privacy of research participants.

The assessment was an ongoing process comprised of four main components: 1) a scoping process with the donor, project partners and key local actors; 2) an in-country feasibility assessment; 3) coordination with local stakeholders and formation of a local TAG; and 4) monitoring of the implementation of the research. The main considerations and challenges that were made during each component are described below.

####  A scoping process with the donor, project partners and key local actors

The initial proposal of the study was presented by a consortium integrated by GWI, IRC (both UK and South Sudan offices) and CARE UK to the donor in early 2013. As a newly independent state, South Sudan lacked solid data on the different forms of VAWG had suffered both before during and after the conflict. Following the proposal, preliminary discussions with governmental institutions, local women’s groups and international actors indicated that such data would be valuable for informing national and international policy and programs. Given the complex setting, these discussions—which commonly take time, effort, and contextual knowledge—were coordinated by IRC. It was essential to have a well-known and respected organization on the ground leading these discussions and facilitating this process.

The study need was confirmed by a desk review that included existing research and other relevant documents to identify existing gaps in evidence. A comprehensive desk review was conducted with the support of national organizations working on VAWG. In all the studies reviewed, VAWG—and non-partner sexual violence in particular—was identified as a major problem in South Sudan; however, no studies could accurately characterize the extent of the different types of violence that women and girls suffered during the various conflict periods.

#### An in-country feasibility assessment

At the project’s inception, the security context in South Sudan was relatively stable; however, with the outbreak of the 2013 Crisis, the context of the study changed considerably. Within this rapidly shifting context, it was critical to assess if it was still feasible and ethical to carry out rigorous research on VAWG. During the feasibility assessment, the research staff conducted meetings with representatives from United Nations (UN) agencies, the local Gender-based Violence (GBV) Sub-cluster, civil society groups and representatives of relevant government ministries, in addition to conducting field visits to possible research sites. All actors who were consulted expressed strong support for the research and they suggested continuing with the study.

Originally, the population-based household survey was planned to be representative of the entire country. Based on the feasibility assessment, it was clear that this was not possible mainly because there were areas affected by the 2013 Crisis that were too risky for the fieldworkers, and this type of survey was not a priority during an acute conflict phase. For that reason, criteria were developed to select study locations that fulfilled minimum ethical standards such as access, availability of referral services, and safety of research staff and participants, as well as typical research considerations such as the adequate representation of sub-populations within the study population.

For those sites that were deemed essential for adequate demographic representation but where a household survey would not be safe or feasible, the research team decided to carry out only qualitative research. The findings from this feasibility assessment were used to populate the risk-benefit analysis, which also informed decision-making on the design and scope of the study to reduce any potential risks as much as possible.

As described above, the survey was only representative of three sites for women (Juba, Rumbek, and the Juba POCs) and two sites for men (Juba and Rumbek). The other sites where only qualitative research was possible to conduct were Bentiu and Juba County. The research team struggled with the dilemma of obtaining important data to show the reality of women’s experiences of violence in the main settings affected by conflict without being able to guarantee safety, or having a smaller representation of the women affected by conflict but making sure that the fieldwork was conducted in relatively safe places. Both were ethically important, however, the research team decided to prioritize the safety of people involved in the research. The limitation of having done the survey in these specific locations was acknowledged in the analysis and the presentation of results, which clearly established that the quantitative data is representative of these places and not the entire country.

####  Coordination with local stakeholders and formation of a local technical advisory group

From inception, the study was conceptualized to be participatory. Key local partners were engaged to help align the research priorities and the priorities of VAWG networks in the region, confirm that appropriate research questions were being asked, and ensure findings would be utilized to inform relevant programs and policies that benefit women and girls. In addition, working with these partners helped to obtain a more accurate assessment of the risks associated with the study and to develop adequate ethical and safety measures that could be put in place to minimize them.

The IRC offices in South Sudan coordinated the involvement of local partners. In addition, a local TAG was established to provide technical support throughout the project timeline, including reviewing and providing feedback on the study protocol, data collection tools, the interpretation of research findings, and dissemination methods for various findings. The TAG was composed of representatives from the United Nations Mission, international and local civil society groups, governmental authorities, and other key South Sudanese actors in the field of VAWG. Partnering with local institutions also enabled the study to link with pre-existing referral networks of accessible services, including psychosocial and health support for women and girls who had survived violence. This ensured that the research participants could be referred to these services throughout the entirety of the study, if needed. Determining that these services were available was essential to confirming whether the study would be feasible in the proposed locations, as it ensured that the risk to participants could be reduced.

In South Sudan, one of the main challenges around involving different local actors was the current conflict between the government, mainly representing one ethnic group – Dinka -, and the rebel armed group mainly representing another ethnic group – Nuer -. The rebel armed group had a strong presence, including being the main local authority in some areas. For that reason, it would have been important to include them as part of the TAG. However, due the sensitivity of the political/ethnic conflict between the government and this group, formal representation of the rebel group in the TAG was not possible. Instead, people from the same ethnic group – Nuer - but without a political affiliation were represented in TAG to ensure adequate representation from all actors.

#### Monitoring of the implementation of the research

The activities that informed the risk-benefit assessment were continually conducted and reassessed throughout the duration of the study. This continual reassessment proved essential when the civil conflict re-ignited during an outbreak of violence in Juba in July 2016, in the middle of ongoing data collection. In the lead up to and during this outbreak in conflict, the consortium partners held regular discussions about the risks and benefits of stopping data collection versus continuing. In mid-July when it became apparent that the conflict was newly acute, the partners decided to pause data collection in Juba City and the Juba POCs. Operations were able to continue in the study site in Rumbek, as the conditions were more stable. After recurrent security and risk assessments conducted over several months, research was resumed in the Juba POC sites in November 2016 and was completed by December 2016. The consortium partners determined that data collection would not be resumed or completed in Juba City due to concerns for the safety and security of both the participants and the enumerators. This continuous risk-assessment was extremely important to discuss alternative scenarios and back-up plans on a regular basis, and to make decisions quickly and effectively when the security situation escalated and required adjustments to the original work plan.

While these assessment activities took a significant amount of time and resources, the results yielded essential information for subsequent phases of the research and helped minimize risk for the people involved in the study. However, the decision to prioritize security during the collection of data had methodological implications. In Juba POCs where the research was resumed after several months, the research team had to go back to conduct refresher training sessions for the enumerators and to make sure the original sample frame was maintained. The goal was to reduce the bias of the survey as much as possible considering the situation. The data collection resumed where the research team left off with incomplete clusters. For clusters in progress, a question was added about previous participation in the household questionnaire. If the selected household was already surveyed, then the fieldworkers moved to the next one. Women who recently arrived to the camp as part of the displacement of the outbreak were not included in the survey in order to keep the original sample frame. However, a recently displaced subgroup of women were included in the qualitative sample to make sure that their experiences were captured. In Juba City were the survey could not be resumed, the sample frame was smaller than originally planned. The sample was still representative of the population of Juba City but had less statistical power.

### Methodological and conceptual approaches

The WHO guidelines outline that research on VAWG in emergencies must be methodologically sound and build on best practice while also presenting the least risk to participants. However, this can be difficult to guarantee in a volatile conflict setting. The constantly changing security context in South Sudan required identifying not only sound methods that could adequately test the research hypotheses, but also a methodology that was sufficiently flexible to adapt to this complex and fluid context. This required a collaborative, iterative approach to the research design process involving both local stakeholders and external experts on research in conflict settings.

Many humanitarian settings are complex and involve multiple levels of conflict and crisis. Ideally, conflicts, types of violence and the temporality of events are defined at the outset of a study to adequately reflect the reality of women and girls’ experiences. VAWG is experienced in many different forms during periods of conflict; nevertheless, the international community and media often focus heavily on conflict-related sexual violence, creating the impression that strategic rape is the most prominent and serious form of VAWG that affects women and girls [10]. For this reason, it was important for the study in South Sudan to have a more expansive view of conflict-related VAWG to include the different types of violence that affect women and girls. The types of violence prioritized in the study were identified and defined in collaboration with the TAG and other local stakeholders. They included physical, sexual, emotional and economic intimate partner violence (IPV); non-partner sexual violence including rape, attempted rape, unwanted touching and sexual harassment; transactional sex; abduction; and harmful discriminatory practices including early and forced marriage, bride price and wife inheritance.

In addition, the study examined the effects of three main armed conflicts identified by the TAG and the local partners: the Sudanese Civil War, the 2013 Crisis in South Sudan, and the on-going inter-communal conflict. The temporality of these conflicts was established using main events that were also relevant locally, such as the Comprehensive Peace Agreement in 2005 marking the end of the Sudanese Civil War, South Sudan Independence in July 2011, the start of the 2013 Crisis in December 2013, and the peace agreement signed in August 2015 [2]. Feedback gathered from key informants during formative data collection was essential for establishing this timeline. This collaborative approach to defining research concepts enabled both the methodology and the findings to be contextually relevant and easily understood by local stakeholders and affected communities.

Another challenge was defining the age group of the study population. Most of the surveys on this topic include participants from 15 to 49 (or sometimes 64) years old. This includes minors between 15 to 17 years old. Including minors in research requires special considerations and protections due to their diminished autonomy. After consulting with global and local experts, the decision was to include this group in the survey but not in the qualitative in-depth interviews. Evidence shows that many cases of VAWG, especially sexual violence, occur in early ages, so it is was important that the research captured information that reflected the current situation of violence that adolescent girls were experiencing.

The WHO guidelines recommended additional safeguards if children are part of the study population. For that reason, the team also took into account other guidance documents during the design of the study and during data collection. These included the recommendations established in the *Ethical Research Involving Children (ERIC)* project compendium with UNICEF [11], and the Population Council’s *Ethical Approaches to Gathering Information from Children and Adolescents in International Settings: Guidelines and Resources* [12], which both focus on ethical considerations specific to research with children and the dynamics between researchers, children, families, communities and other stakeholders.

Many adolescent girls were already in a marriage union in South Sudan, so it is common that they already experienced IPV. Those unmarried adolescent girls who were still living in the home of the family of origin and suffered sexual abuse often experienced it at home. In this context, the research team decided to waive parental/adult permission considering that attaining permission from a parent may put the child at further risk in the household. Instead, adolescent girls still living in the home of the family of origin were asked for an informed assent of their participation. Those who were already married and living with a husband were asked for an informed consent.

In addition, only adolescent girls aged 15–17 were included if they showed ability to understand the nature of the research and were capable to provide informed consent. During the training of the data collectors, facilitators suggested to go particularly slow with adolescent girls ages 15–17 in order to explain the nature of confidentiality, consent and the voluntary nature of their participation in the research. Consent and assent forms for both the quantitative survey and the qualitative interview were read to the respondents and used simple language that could be easily understood by adolescents. All respondents had a chance to ask questions of the data collectors at any time before, during, or after the interview. No particular challenges arose from the inclusion of this group in the research. More information on data collector training to minimize risks to participants can be found in the below section on Safety considerations.

The next challenge was to develop tools that capture the experiences of violence that women suffer and their association with the different types of conflict. For the cross-sectional survey, the first step was to conduct a scoping exercise to identify existing tools relevant to the research questions. A detailed matrix was developed to analyze surveys on VAWG in conflict and humanitarian settings from a variety of sources. The matrix supported the development of the household questionnaires for women and men and ensured that they were grounded in tested methodologies. In addition, the research team collaborated with other researchers working on sensitive topics in conflict and humanitarian settings to review best practices and to refine the final tools that were utilized in South Sudan. The questionnaires were based on the WHO multi-country study on VAWG questionnaire, but adapted for a conflict and humanitarian setting. The WHO study is recognized as one of the most validated models when doing quantitative research in the field. The questionnaires were finalized with the support of the TAG and local IRC staff who reviewed them for relevancy and cultural appropriateness. The qualitative tools followed a similar process.

A second step was to conduct formative research using participatory action methods. This formative research included primarily semi-structured in-depth interviews with key stakeholders and local actors in South Sudan plus participatory focus groups discussions with male and female community leaders and young men and women. The formative research improved the understanding of the overall context of VAWG and conflict in South Sudan, and informed the design of the survey and the qualitative research.

While these methodological considerations are common of all descriptive research on VAWG, the fluid dynamic of humanitarian settings presents an added challenge. When developing the sampling frame, though it was important to utilize sufficient sample sizes to produce rigorous findings, the high-risk environment presented several barriers. As a safety measure to minimize risk for female participants, the research team used separate sampling frames for men and women. This ensured that the study did not draw unnecessary attention or raise suspicion of those taking part, thereby reducing the risk of a breach in confidentiality and helping to ensure the safety of participants. A multi-stage cluster sampling design was therefore used to select individual households for inclusion in the cross-sectional survey, and interviews with men and women were conducted in different clusters. However, maintaining separate clusters for men and women was particularly difficult in POC sites where safe and private spaces were limited. After consulting with local stakeholders and external researchers, the research team decided to exclude men from the sample in the POC sites to reduce the risk to female participants participating in the survey. This had an important implication in the data and analysis meaning experiences of men were not collected, both as perpetrators and victims, in the POCs. However, again, the safety of participants was prioritized.

### Safety considerations

In line with the WHO guidelines, guaranteeing the safety and security of the participants, their communities, and the data collection team should be the upmost priority of any study in VAWG and should be continuously monitored. However, this has a lot of challenges when doing research in a conflict setting. The situation in South Sudan shifted quickly from a post-conflict setting to an active conflict in the middle of data collection, requiring continual assessments to ensure that protections for participants were maintained despite fluctuations in security and other unexpected events. Over time, it became extremely challenging to guarantee the safety of all participants. The underlying ethnic tensions of the conflict, which became even more heightened as it re-escalated, also required additional considerations for the safety of all enumerators.

#### Coordinating with local networks and stakeholders

Working with IRC as an implementing partner with strong local networks was essential to facilitating community engagement, gaining access to key stakeholders and community members and receiving vital security information. IRC security protocols were strictly followed by the research team throughout the entirety of data collection, and the team only conducted research in locations that had these security protocols in place. Current security information was collated from a range of sources and fed to the research team through regular briefings with IRC’s security team. These briefings were integrated into all planning and logistical meetings to ensure that the research team had a clear understanding of the situation on the ground at all times. To have relatively safe access to research sites and the cooperation and protection of authorities at the national and local level, IRC, with the support of the TAG and Forcier Consulting, obtained permissions, or sanctioned agreements to conduct research activities within the community, with the relevant actors.

IRC’s strong presence in the research sites was crucial to the planning and implementation of research in those locations and enabled safe and secure data collection. This was particularly evident when tensions mounted in Juba and violence broke out across the city in the middle of data collection in July 2016. IRC’s security assessments were pivotal to the decisions to stop and start data collection and to discontinue the study in Juba City due to the inability to prevent adverse events.

#### Enumerator selection and training

In addition to security monitoring, it was extremely important to have measures in place to avoid unnecessary harm or discomfort to participants. Respect for persons is one of the core principles of research ethics and is highlighted by the WHO; however, protecting the rights, dignity and autonomy of participants in conflict and humanitarian settings is particularly challenging given the heightened level of vulnerability of the population.

One of the most important activities to increase the protection for survivors was the selection and training of enumerators. Enumerators were carefully selected and trained during an interactive three-week training session held at each site, which included instructions on managing privacy, confidentiality and participant distress, as well as questionnaire and study methodology.

To reduce risk for respondents, fieldworkers were members of the communities in which they were conducting research where possible, and/or they were from the same ethnic group as the individuals being interviewed; therefore, they had a strong rapport within those communities, in addition to speaking the same language and sharing cultural similarities. Careful attention was given to ensuring that interviewers were not exposed to risks due to their ethnic affiliation at any point during training or data collection. At the same time, we made sure that enumerators did not know any of the selected participants that they had to interview. Due to South Sudan’s cultural and ethnic context, it was challenging to find women’s fieldworkers with the characteristics that were needed (high-school educated, previous experience in doing research in sensitive topics, some gender awareness) that represent all different groups.

The training sessions for the fieldworkers needed to be adapted to the specific context of South Sudan. These adaptations were more practical than theoretical and required the use of local specific examples to represent different potential situations. Tools for the training sessions had to be simplified to allow for better understanding. To ensure that the fieldworkers were prepared, a lot of time was dedicated to doing role plays with the research team. Regular debriefs were also held with the fieldworkers to ensure continuous learning, support and improvement; however, sometimes this was difficult due the conditions of the context.

#### Protecting confidentiality and voluntary participation

Extra precautions were taken to follow the WHO guidelines regarding confidentiality and voluntary participation of selected participants and reducing distress or anxiety during or after the interview. Regarding confidentiality, the main challenge was to carry out the interviews in completely private spaces with no one else in view or hearing distance, especially in those settings where participants were in extremely close quarters, such as POC sites. Qualitative in-depth interviews were held in NGO offices and women’s safe spaces to guarantee privacy. For quantitative interviews, if the privacy of the survey respondent could not be assured when in their home, interviews were moved to rooms in community centers, NGO offices or other pre-determined safe spaces. However, there were situations where total privacy could not be assured, in particular in the POC camps where relatives and friends lived in cramped and close quarters. In these cases, enumerators were trained to try to re-schedule the interviews; however, in some cases they were not able to guarantee complete privacy and the interview was not conducted. Although these cases were small, it may have introduced a bias by not interviewing those women who could not be in total privacy for the survey.

Regarding voluntary participation, the power imbalance between these vulnerable populations and the organizations who conduct research—especially when an implementing partner is involved—can make participants susceptible to influence to participate due to fear of retribution or loss of services if one decides not to participate. There was special attention to this potential power imbalance during the training to fieldworkers. Particular emphasis was put on how to introduce the study. The IRB requested to have a large and detailed informed consent form to read to participants. In this context, this mechanism did not work because many women in South Sudan are illiterate and they are not use to listen to long and complex document. Instead, fieldworkers provided, with their own words, a very detailed explanation of the purpose of the study, the potential risks, how the information was going to be used, and the long-term benefits for the community. Many hours were spent during the training to enumerators practicing the introduction of the study.

In addition, fieldworkers encouraged respondents to ask any questions or voice any doubts or concerns before and during the interview and they tried to give appropriate answers to all questions. Participants were advised that some of the topics would be extremely personal and may be difficult to talk about. Participants were informed at the beginning of the interview and reminded throughout that they could choose to skip questions or stop their participation at any time if they no longer wished to participate.

In all cases, the aim was to obtain this permission without the use of any coercion or inducement or promising unrealistic benefits for the participants. However, despite all these efforts, it is not clear at what extent participants accepted to be part of the study because they felt obligated to do it or because they felt that they were going to obtain a direct benefit from it. Consent was obtained verbally, as obtaining written consent would not be appropriate given the large percentage of the population that is illiterate.

#### Minimizing distress to participants

To minimize distress during and after the interview, basic care and support for survivors of violence, as well as other participants needing psychosocial support, were in place before beginning data collection. A list of resources for survivors of violence was developed for referral to quality services in each site in South Sudan, including health, psychosocial, women’s support and legal/police. This list was offered to all participants regardless of whether or not they reported experiencing violence. In addition, survivors of violence who participated in the in-depth qualitative interviews continued to receive services from IRC as part of on-going GBV case management.

Due to the high level of trauma in this setting, significant efforts were also made to minimize re-traumatization for participants. The field workers were trained to identify signs of distress; if a respondent appeared to be in distress or experiencing re-traumatization, they stopped the interview and allowed the respondent to compose herself/himself. The enumerators then asked the respondent if she/he wished to continue or to stop the interview and talk to a trained counselor. Psychosocial support workers were available with vehicles in all sites to visit participants in their homes if they experienced significant distress during the interviews. In addition, service providers at IRC and other local organizations were advised to ask women who visited their centers as a result of referral from the study (identified by presenting the referral card distributed at the end of the survey) whether they experienced additional violence as a result of their participation in the study. The survey tools also included a final question (“How has talking about these things made you feel?”) to track the percentage of participants who felt worse after participating in the survey. This measure was monitored throughout the duration of data collection and the percentage was consistently extremely low.

#### Vicarious trauma

This study also acknowledged the risk of vicarious trauma for researchers and fieldworkers. Researchers were often exposed to personal and sometimes upsetting accounts of participants’ experiences of violence. While emotional engagement can be a tool for researchers to provide a safe and comforting environment for participants, it can also carry an emotional cost for the researcher. As part of this research, fieldworkers and research staff were provided with on-going support, including basic psychosocial care, regular check-ins, and weekly debriefing sessions throughout the duration of data collection to minimize any distress on their part as a result of listening to participants’ experiences of violence. However, sometimes it was very difficult to provide this support. Operational and logistics challenges to transport the research team, especially during community attacks, or insufficient capacity for case management services (which prioritized research participants or other cases) were among some of these challenges. For that reason, some sessions related to self-care were included during the training.

### Analysis and research uptake

After data collection was completed and results were analyzed, it was important to ensure that the findings from the study would be utilized to improve the lives of women and girls. The presentation of findings from VAWG research is sensitive no matter the setting; however, in South Sudan, political and cultural tensions made it especially difficult to present data without interfering in the conflict or having negative consequences on those involved in the research. Therefore, the research consortium developed several different research products as part of the uptake phase. In addition to a full report [2], which could be immediately disseminated to global stakeholders, the research team developed a policy brief, presentation materials targeted at communities, and academic papers. With different audiences, the presentation of findings in each of these products was slightly adjusted to ensure that they would be relevant to specific audiences and to minimize potential risks to participants and their communities.

In accordance with ethical standards, identifying information was removed from all reports to protect the confidentiality of all research participants. After determining the audience for each research product, further consideration was given to potentially harmful information and whether some findings might breach confidentiality in specific contexts if presented to local stakeholders. Therefore, additional identifying information including specific location (for example, the specific POC site in Juba), political affiliation and ethnicity were also removed.

While academic papers and reports published at the global level included specific reference to research sites without causing harm, presenting this level of information at the local level could have significant negative consequences. To avoid causing harm to communities or fueling tensions between parties in the conflict, research findings were presented with care and in as neutral a way as possible. Given the ethnic nature of the conflict in South Sudan and the potentially oppressive behavior of some national actors, particular consideration was given to findings that might incite further violence between participating communities. After consultation with the TAG and local IRC staff members, certain references to politically sensitive topics were removed; for example, removing the specific affiliation of armed combatants when mentioned as perpetrators of violence. Additionally, as geographic locations are strongly associated with specific ethnic groups, care was taken when presenting population-specific statistics to local audiences, and more general figures without reference to ethnicity were utilized. While the presentation findings around specific perpetrators raised ethical questions as removing reference to specific perpetrators may have reduced accountability, the safety of participants took precedence and so findings referring to specific groups were de-identified.

Involving local stakeholders in the research uptake phase was key to ensuring that findings would be used while also reducing risk to participating individuals and communities. The TAG, GBV Sub-cluster and local staff from the consortium partners had the opportunity to provide input on analyses and drafts of the report, confirming that the findings were relevant. The TAG and local IRC staff also supported the dissemination of findings through their relevant networks, guaranteeing that the research would help to improve the lives of women and girls in South Sudan.

Since the time of the study, findings have been shared at the international level in a number of high profile events including at the United Nations. In addition, results have also been presented in South Sudan to the TAG, local governmental and non-governmental organizations, and UN agencies, among other key institutions. These presentations were conducted in addition to a workshop for local actors, where representatives learned how to interpret the data in the report and made plans regarding how to put this research into action. In addition, the study has been featured in multiple international and national media outlets.

Specifically, in order to reach community members where the research was conducted, a data-to-action process was planned with local stakeholders in South Sudan and has begun to be implemented. This includes the development of a workshop with local women’s organizations, artists, and other key stakeholders to develop together participatory program tools using the data and stories collected in the study. Artists will assist with the design of the tools in order to translate the research results and stories into visual tools that can be used by the stakeholders.

## Discussion

Any research involving human subjects must consider ethical standards developed and validated by the scientific community. The South Sudan study confirmed that the application of universal ethical standards can present several difficulties, especially in conflict and humanitarian settings, some of which can cause important dilemmas for the researcher. The response to these dilemmas can be very complex and may require an in-depth ethical and methodological analysis.

The WHO guidelines provide an important framework for conducting research on VAWG in conflict and humanitarian settings. But, what are the main challenges in the application of these guidelines? And do these guidelines sufficiently take into account the ethical, methodological and safety challenges for conducting research on VAWG in conflict and humanitarian settings? The following section outlines reflections on each of the WHO guidelines based on the South Sudan study.

### The benefits to respondents or communities of documenting sexual violence must be greater than the risks to respondents and communities

Many activities were carried out to make sure that the research was needed. However, implementing these activities requires a lot of time and resources, both human and financial. This can be particularly challenging during an acute conflict. Donors, researchers, programmers, and all key actors involved in a study like this should be aware of the importance of this assessment, as well as the financial and operational implications of this process to make sure that the research is needed and will benefit women and girls.

### Information gathering and documentation must be done in a manner that presents the least risk to respondents, is methodologically sound, and builds on current experience and good practice

Similar to ensuring the needs and benefits for research on VAWG in conflict, deciding on a strong and appropriate research methodology takes time and has financial implications. For this research in South Sudan, it was vital to conduct initial review around what others have done globally and locally on the same topic. Although it was important to be innovative and adapt methodologies to this particular research, there was no need to re-invent the wheel.

While some rigorous methods may not be feasible in certain settings, this research benefited from utilizing mixed quantitative and qualitative research methods because it enabled an in-depth exploration of VAWG in a complex crisis setting. This was particularly important considering that it was the first study in South Sudan that attempted to determine the levels of different types of VAWG and their connection with the conflicts.

One of the main methodological challenges of this research was defining the need for a population-based household representative survey. This type of survey provides an estimated diagnosis of the magnitude and explanation of the factors, consequences, and circumstances of VAWG in representative areas. By providing a clearer picture of how different types of violence are connected to different conflicts, settings and phases of a crisis, quantitative findings can be generalized to other similar conflict and humanitarian settings, and can be used by VAWG actors to prioritize specific types of violence at specific times in a crisis. However, conducting this type of survey can be methodologically and ethically challenging and the caring of the WHO considerations could be very costly and with a lot of operational and logistics implications. While deciding whether or not a population-based survey is necessary, researchers should consider the need, previous surveys, resources, logistic implications, and the phases of the crisis (acute, protracted or post-conflict). While it is important to capture this information, a population-based survey might be a better choice during a more stable phase of the conflict.

Another important methodological implication in this study was the establishment of key concepts such as the types of violence and the types of conflict that will be analyzed in collaboration with local partners. In conflict settings, many types of VAWG can be present in one setting, as well as different types of conflict. The situation in South Sudan is very complex and involves multiple levels of conflict and crisis. In this research, it was challenging to distinguish between periods of conflict and peace in order to establish temporality.

The documentation of results deserves specific ethical guidance when doing research in conflict and crises. Ethically, the research team felt responsible to conduct the best possible actions to ensure that the research findings benefited communities and were presented for different audiences in a way that makes the findings easy to uptake for policies and programs.

In conflict settings such as South Sudan, political and cultural tensions can make it difficult to present data without interfering in the conflict or having negative consequences on those involved in the research. For example in this case, government and some local actors are opposed to one another, making the presentation of findings particularly challenging. While involving community members in the dissemination process is very valuable, there is also a risk of bias in politically polarized environments or environments where ethnic tensions are high. The publication of research findings in oppressive settings could even cause problems for the researchers or the organizations involved in the research.

Additionally, lack of governance in humanitarian settings can make it difficult for actors to sufficiently implement any recommendations or changes that arise from the research. In the case of South Sudan research, presenting the findings in a confidential way was important to reduce potential risks. Additionally, to avoid causing harm to communities or stoking tensions between parties in the conflict, research findings were carefully presented in the most neutral way possible. Finally, different dissemination formats were utilized for different audiences so as to ensure both adequate understanding and also to avoid eliciting negative reactions. Having designated funds for the development and implementation of a research uptake plan from the outset of the study was key.

### Basic care and support for survivors/victims must be available locally before commencing any activity that may involve individuals disclosing information about their experiences of violence

The research team took this consideration very seriously, at the extent that one of the criteria for selection of the research setting was the feasibility to have services available where the data was collected. The presence of IRC and the accompanying services were extremely important for this research in order to fulfill this consideration. However, this introduced a clear methodological limitation, as the research was only conducted in locations where services were available. The experiences of women where no services were available were left out of the scope of this research.

### The safety and security of all those involved in information gathering about violence is of paramount concern and in emergency settings in particular should be continuously monitored

The research in South Sudan prioritized this recommendation over all of the other ethical considerations. This caused several ethical dilemmas where the research team had to decide between the protection of the safety of participants and fieldworkers and the reduction of methodological limitations and bias to the study. However, putting participants and staff at risk was not an option, and for that reason all possible mechanisms were put in place to protect everyone involved in the research. This includes adherence to the security protocols and operational preferences of implementing partners, holding regular meetings about security, developing back-up and flexible plans, and pausing data collection during acute conflict. The volatile nature of this particular conflict required close and constant security monitoring. Similar to the other considerations, this took time and effort for the people involved in the research.

### The confidentiality of individuals who provide information about violence must be protected at all times

Confidentiality is a key consideration to avoid harm, stigma, or discomfort to respondents. In the context of this research, informal living conditions made it more difficult to maintain confidentiality - especially in the POCs. If privacy could not be established in the home of the participant, extra precautions were taken, such as using different venues like community centers, NGO offices and other buildings. All interviews were conducted in private spaces.

### Anyone providing information about violence must give informed consent before participating in the data gathering activity

Several scholars have stated the difficulties around obtaining true consent for participation in research, especially in contexts with marginalized and vulnerable population. The voluntary participation was a serious consideration taken by the research team. For that reason, informed consent procedures were adapted to the local context. However, in humanitarian contexts like South Sudan, there can be false expectations of individual study benefits that may encourage participation. Some participants may have agreed to participate because they assumed that this would be positive – or at least not negative - for them or because they felt obligated to participate. The fieldworkers tried to avoid these situations, and in all cases, tried to have an equal relationship with participants and to obtain permission to conduct the interview without the use of any direct or indirect coercion, inducement or by promising unrealistic benefits. However, despite of these efforts, it is not clear to what extent participants agreed to be part of the study in a totally voluntary way.

### All members of the data collection team must be carefully selected and receive relevant and sufficient specialized training and ongoing support

As previously mentioned, the selection and training was extremely important for the successful conduction of the survey. However, both the selection and training were very challenging in a context such as South Sudan. In conflict and humanitarian settings it can be very difficult to carry out a long training considering the conditions. In the future, other innovative approaches to training could be explored to ensure efficiency, while making sure fieldworkers receive the basic information needed to conduct the research in an ethical and rigorous way.

### Additional safeguards must be put into place if children (i.e. those under 18 years) are to be the subject of information gathering

In this research, the decision was made to include individuals aged 15 to 17 in the survey. This was approved by the TAG and the local researchers and programmers. Extra measures were taken to protect the participants that fell under this category. In addition, the decision was made not to include adolescent girl survivors in the qualitative in-depth interviews to reduce the risk of possible distress among adolescent girls.

In sum, the WHO Recommendations provide an important framework to carry out research on VAWG, taking into account main ethical considerations. However, we consider that this will improve if a distinction is made between ethical principles and the means to address these principles. Ethical principles in sensitive research are those based on the Belmont Report related to the respect, benefit and justice to all people involved on research. In this case, issues such as security, avoiding harm, consent, and benefit for the community would be ethical principles. Instead, guaranteeing confidentiality, training of enumerators, and provision of referral services are a means to address these issues. This distinction is important to help researchers to better organize their thoughts on how to address these ethical issues. The WHO ethical recommendations would also benefit from providing more practical means to addressing ethical dilemmas when doing research on VAWG in conflict and humanitarian settings, rather than just theoretical concepts.

In the conflict and humanitarian field, efforts have been made to address some ethical considerations when doing research with human subjects in general. For example, the Medicines Sans Frontieres (MSF) developed an ethical framework for conducting research in conflict settings [10]. The framework presents nine benchmarks for the ethical conduct of research. Several of these benchmarks are included in the WHO recommendations, however, they further emphasize the importance of community engagement and having collaborative partnerships with local actors. Another consideration is about the fair selection of participants, given that in conflict areas—especially within refuge and displacement settlements and camps—there is research fatigue among the population because those settings are more accessible for research.

It is very important to have a specific framework when doing research on VAWG in conflict and humanitarian settings, but it is important to keep in mind that the researchers will face some ethical dilemmas that contradict some of the ethical recommendations. For that reason, the framework should be flexible but at the same time should provide specific practical recommendations of how to address ethical challenges should they arise.

While doing research on VAWG in conflict and humanitarian settings, there is a clear tension between the need to conduct research to seek the well-being of the society and the respect and safety of the participants and their communities. This balance needs to be continuously assessed by researchers, and more innovative methods needs to be developed to reduce risks while doing research in these types of settings.

Finally, due to the challenges presented by the situation in South Sudan, it is necessary for all partners and members of the research team to adapt their expectations around logistics and what is feasible within timeframes. All aspects of the research process, including moving the entire research team to each location, were much slower than expected in this conflict setting.

## Conclusions

Ellsberg and Heise [13] stated that research on VAWG can be done with full respect for ethical and safety considerations if proper care and resources are devoted to this end. This is extremely important in conflict and humanitarian settings where women are already at risk. All researchers involved in studies of this topic, in these settings, have the ethical obligation to take every precaution possible to minimize risks and maximize benefits. Important efforts are needed to improve the capacity of researchers and practitioners to adequately address ethical and methodological issues when undertaking research in conflict and humanitarian settings. In addition to following international guidelines such as those set forth by the WHO, all research on conflict and humanitarian settings should be reviewed and approved by an ethical review board, and every ethical review board should have members who possess knowledge of human subjects’ research on sensitive topics in high-risk settings.

The study in South Sudan provided an excellent opportunity to put into practice the international ethical guidelines to carry out research on VAWG in conflict and humanitarian settings. The study enabled the research team to reflect on the guidelines and develop strategies to cope with new methodological and ethical challenges that arose in this complex setting, adapting the guidelines, as necessary. It revealed the importance of developing a strong yet adaptable methodology and multiple alternative plans in order to efficiently and effectively solve any safety or ethical issues that might occur throughout the entirety of the study. It also emphasized the importance of collaborating with an implementing partner and gathering input from both local and international stakeholders to ensure a sound, relevant methodology and effective research uptake activities. The primary lesson learned derived from the study in South Sudan was that this type of research is extremely complex and necessitates significant planning, in addition to substantial financial and human resources. Since its completion, the study and its methodology have been used as models for conducting rigorous research on VAWG in emergency settings.

A key component of the success of the study in South Sudan was the social commitment to conducting research on VAWG in such a challenging context in an ethical and rigorous way. The buy-in and commitment of not only the consortium partners but also the donor, local stakeholders, and external experts and organizations were essential to the safe and successful completion of the study. Research is a social commitment and all actors involved should not forget the ultimate goal of carrying out research on VAWG in conflict and humanitarian settings: to improve the lives of women and girls. This goal must be the foundation of all research on VAWG and should inform all of the multifaceted ethical considerations and decisions that are made during a study to further protect women and girls.

## Data Availability

Report of the results of the South Sudan study can be found in the following link: http://www2.gwu.edu/~mcs/gwi/No_Safe_Place_Full_Report.pdf

## Abbreviations

CIUK: CARE International UK
CPA: Comprehensive Peace Agreement
DfID: Department for International Development
GBV: Gender-based violence
GWI: Global Women’s Institute
IPV: Intimate partner violence
IRC: International Rescue Committee
MSF: Médicines Sans Frontières
POC: Protection of Civilian
TAG: Technical Advisory Group
UN: United Nations
VAWG: Violence against Women and Girls
WHO: World Health Organization
